# Proteomic analyses of Urine Exosomes reveal New Biomarkers of Diabetes in Pregnancy

**DOI:** 10.18689/mjd-1000103

**Published:** 2016-02-01

**Authors:** Satish P Ramachandrarao, Alyssa A Hamlin, Linda Awdishu, Rachael Overcash, Marcela Zhou, James Proudfoot, Michelle Ishaya, Eamon Aghania, Assael Madrigal, Chanthel Kokoy-Mondragon, Kelly Kao, Roni Khoshaba, Anousone Bounkhoun, Majid Ghassemian, Maryam Tarsa, Robert K Naviaux

**Affiliations:** 1 Department of Medicine, Biomarkers Laboratory, O’Brien Center for Acute Kidney Injury Research, UC San Diego, USA; 2 Department of Pediatrics, Center for Promotion of Maternal Health and Infant Development, UC San Diego, USA; 3 Department of Reproductive Medicine, UC San Diego, USA; 4 Clinical and Translational Research Institute, UC San Diego, USA; 5 Department of Chemistry & Biochemistry, Biomolecular & Proteomics Spectrometry Facility, UC San Diego, USA; 6 Departments of Medicine, Pathology and Pediatrics, UC San Diego, USA

**Keywords:** Damage associated molecular pattern, Diabetic pregnancy, Exosome, Gestational diabetes, Urine exosomes, Proteomics, S100A9

## Abstract

**Objective:**

To evaluate 24 hour urine exosome protein content changes among pregnant US subjects with diabetes and obesity during early pregnancy.

**Methods:**

The exosome proteome content from 24 hour urine samples of pregnant subjects with gestational diabetes mellitus (GDM, N=8) and pre-gestational Type 2 diabetes (PGD, N = 10) were compared with control samples (CTRL, N = 10) obtained at week 20 of pregnancy. Differences in exosome protein load between groups was identified by liquid chromatography/mass spectrometry, analyzed by linear regression in negative binomial distribution, visualized in MetaboAnalyst (version 3.0), and validated by western immunoblotting.

**Results:**

At the 20^th^ week of pregnancy, we identified 646, 734 and 856 proteins in exosomes from 24 hour urine samples of patients from the CTRL, GDM and PGD groups, respectively. S100 calcium binding protein A9, damage associated molecular pattern (DAMP) signal, was found to be significantly increased in both GDM and PGD subjects. In GDM subjects the peptide counts for S100A9 protein independently correlated with maternal obesity and macrosomia of the newborn infants. Early to late pregnancy developmental changes in the GDM group were shown to utilize pathways and protein expression levels differently from those in PGD or CTRL groups.

**Conclusions:**

Urinary exosome proteomic analysis non-invasively provides insights into maternal changes during diabetic pregnancy. Exosome biomarkers early in pregnancy can be potentially used to better understand pathophysiologic mechanisms of diabetes at a cellular level, and to distinguish between gestational and pre-gestational diabetes at the pathway level. This information can aid intervention efforts to improve pregnancy outcomes in women with diabetes.

## Introduction

Diabetes during pregnancy increases the risk of poor pregnancy outcomes. Poorly controlled maternal diabetes may increase the risk of malformations involving multiple organ systems, such as fetal cardiac and spinal abnormalities during the first trimester and even fetal loss during the third trimester [1]. Renal glomerular filtration rate and plasma flow increase during normal pregnancy, leading to increases in proteinuria, glucosuria, and lower serum osmolality and sodium levels [2]. In pregnancies complicated by diabetes these changes may be exacerbated causing subclinical renal insufficiency in addition to the other unfavorable outcomes mentioned [3]. Pre-gestational diabetic (PGD) mice develop accelerated renal pathology [4], suggesting that stimuli that elicit benign responses in the non-diabetic pregnancies may lead to renal dysfunction or injury in diabetic pregnancies [5,6]. Further, as demonstrated by the collaborative perinatal project, the PGD mothers carry different risks of giving birth to malformed infants than do the GDM mothers [7]. Mechanisms underlying end organ damage or malformation and the maternal/fetal metabolic programing affecting the kidney function of the newborn still remain incompletely defined [8–11]. New tools that are noninvasive yet specific enough to examine the pathologic alterations in maternal diabetes during pregnancy are required to study clinically relevant sequelae specific to GDM or PGD phenotypes. We believe that urinary exosomes represent a noninvasive paradigm that potentially may be able to address phenotype-specific questions.

Exosomes are membrane bound biologically active nanovesicles released from every cell type [12,13]. They are formed by inward budding of late endosomes, producing Multivesicular bodies (MVBs). They are then released into the surrounding biofluid by fusion of MVBs with the plasma membrane [14]. The endocytic origin of exosomes and the cellular nature of their contents enable them to be recognized as a rich source of information on the status of the cells producing them. Because their contents significantly change in response to stress or injury [15] they are also potent mediators of intercellular communication [16]. Average human kidneys filter 1500–2000 liters of plasma on a daily basis to produce roughly 1.5 liters of urine. Together with the observation that urinary exosomes contain 3–5% of the total urinary proteins, exosomes represent enriched background information on the subject’s systemic status.

Diabetic pregnancy is a stressor on multiple organs, including the kidney. Further, GDM is qualitatively a different type of stress compared to PGD [7]. Therefore, at an early time point during diabetic and normal pregnancy we sought to characterize urine exosome proteins from CTRL (n=10), PGD (n=10) and GDM (n=8) women. We also investigated pathway level differences between pre-gestational and gestational diabetes, as well as developmental changes from early to late pregnancy as reflected in their urine exosome proteomes.

## Materials & Methods

### Patient Selection

Singleton pregnancies who received prenatal care at the University of California San Diego Medical Center from 2011 to 2013 were consented to participate in the study. The study design and recruitment was reviewed and approved by the institutional review board. Informed consent for participation was obtained from all study participants. Inclusion criteria included pre-existing Type 2 diabetes or gestational diabetes. Diagnosis for gestational diabetes followed the criteria established by the California Sweet Success Program. This criterion includes at least one of the following: a first trimester HbA1c between 5.7–6.4% (39–46 mmol/mol), a fasting plasma glucose between 92–126 mg/dL, or a second trimester two hour glucose tolerance test with a fasting plasma glucose of greater than or equal to 92 mg/dL, one hour greater than or equal to 180 mg/dL, ora two hour plasma glucose greater than or equal to153 mg/dL [17]. Control samples were obtained from consenting women who did not have preexisting diabetes and screened negative for gestational diabetes. Study participants were required to collect a 24 hour urine sample at 20 and 36 weeks gestation.

## Demographics

Data was collected from a chart review. Information regarding diabetes management and control were obtained during first, second, and third trimesters, including HbA1c, fasting plasma glucose levels, diabetes medications (oral hypoglycemics and insulin), and any maternal medical complications. Fetal outcomes including ultrasound, estimated third trimester fetal weight, and birth weight were also collected.

### Study design

Urine samples at the 20^th^ week (early time point) of pregnancy from 8 GDM, 10 PGD and 10 CTRL subjects were used for exosome isolation and proteomic analysis in replicates. A subset of these subjects that completed sample collection during 36^th^ week of pregnancy (5 GDM, 3 PGD and 4 CTRL pregnancy subjects) were separately analyzed to evaluate differences between the 20^th^ and 36^th^ week. Proteomic data were analyzed using negative binomial distribution as well as MetaboAnalyst (version 3.0) and validated by Western immunoblotting where applicable.

## Urine Sampling and Processing

24 hour urine samples were collected spanning 2 days. The first void of Day 1 was not collected. All voids were collected from the second void on Day 1 to the first void on Day 2, including all nocturnal voids. During collection the samples were kept on ice or refrigerated. After 24 hours collected urine samples were centrifuged at 3000 × *g* for 30 min. The pH of the resulting supernatant was adjusted to 7.0, aliquoted, and frozen at −70 °C until further analysis.

## Exosome preparation and proteomic analysis

Exosomes from frozen pregnancy urine samples were prepared using an in-house protocol developed based on the solvent exclusion principle using polyethylene glycol (PEG)- induced precipitation, as described in our recent publication [18]. One-dimensional SDS-PAGE of the exosome proteins prior to in-gel trypsinization was performed.

## Data Processing and Statistical Analysis

In all analyses, *p* ≤0.05 was considered statistically significant. Log transformation of raw data were performed before the tests. Analyses including Student’s t-tests, Partial-Least Squares Discriminant Analysis (PLS-DA) and Variable Importance in Projection (VIP) were performed with the MetaboAnalyst 3.0 (www.metaboanalyst.ca) web portal [19]. Negative binomial generalized linear regression models were fit using the statistical software R version 3.1.2.20

PLS-DA and VIP were used both for the classification and significant feature selection [19] with a False Discovery Rate (FDR) of ≤10 % to filter protein candidates for western immunoblotting validation. Negative binomial generalized linear models were fit to determine significant differences in protein counts between CTRL, GDM, and PGD subjects utilizing the raw count data and estimating gene-wide size factors and estimated dispersion-mean relationship via the methods outlined in Anders and Huber in the R package DESeq2 [21]. Significance was reported based on a filtered FDR-adjusted p-value.

## Western Immunoblotting and Quantification

Antibody against S100 A9 was purchased from Proteintech Group, Inc., (Chicago, IL, USA). HRP-conjugated secondary antibody was from GE Life Sciences (Piscataway, NJ, USA). SDS-PAGE gels (with 10% acrylamide) were used to resolve 100 μg of protein from exosomes of normal and diabetic pregnancy urine samples. Immunoblotting and quantification with Image J software (NIH) was done using methods described in our previous publications [22] and plotted using Graph Pad Prism software (San Diego, CA, USA).

## Results

### Patient Demographics Show Significant Differences between Diabetic and Non-Diabetic Pregnancy Subject Phenotypes

We studied 24 hour urine samples from 28 mothers: 10 non-diabetic subjects that served as controls (CTRL) and 18 with diabetes (8 gestational, GDM and 10 pregestational, PGD) recruited from UC San Diego Reproductive Medicine clinics. Clinical disease data on 6 GDM, 7 PGD and 6 CTRL mothers was collected. Newborn birth weight in PGD averaged 3155.3 gms as compared to 3884.8 gms in GDM group. Medication regimens were different between the two groups: while all 7PGD subjects required medication, only 2 GDM subjects required medication. The level of diabetes control between the PGD and GDM groups was different. For this study, HbA1c ≥7% (53 mmol/mol) was considered a marker for poorly controlled disease. Accordingly, our data indicate that 5 of the 7 PGD patients were poorly controlled, while all of the GDM patients were well controlled. The minimum diagnostic criteria based on HbA1c are 5.7% (39 mmol/mol) for GDM and 6.5% (48 mmol/mol) for PGD. In our cohort, the average HbA1c was 6.0% (42 mmol/mol) for GDM and 7.1% (54 mmol/mol) for PGD subjects, showing an average increase of only 0.053% above the diagnostic criteria for GDM, but an increase of 0.09% above the diagnostic criteria for the PGD group. Thus GDM group subjects had better controlled disease than PGD subjects, possibly due to the shorter time course of GDM as compared to PGD (Table 1).

Average maternal BMI and neonate head circumference were similar between the GDM and PGD groups (Table 2). None of the PGD neonates in our cohort was macrosomic (average birth weight 3155.3±664.5 gms). Of the 6 GDM neonates, 3 were macrosomic (4485±86.07 gms) and 3 were normal (3285±337 gms). Thus phenotypically, both the mother and the newborn infant in the GDM group were different from that in the PGD group.

### The Urinary Exosome Protein Content is Different in Diabetic *versus* Normal Pregnancy Subjects

Urine exosome proteomic data from an early time point of pregnancy (24 hour urine samples from week 20) showed that 1103 proteins were identified with a spread of 645 CTRL proteins, 855 GDM proteins and 733 PGD proteins in each subject of the group.475 proteins were common to all three groups (Figure S1). Tables S1–S5 document the individual protein identity of each of the groups. These protein data show differences in the non-diabetic *versus* diabetic pregnancy urine exosomes’ protein signatures, and further differences between GDM and PGD subjects at an early pregnancy time point.

To further delineate semi-quantitative differences between groups we used Negative Binomial Distribution to perform linear regression of the expression data of 1103 proteins. CTRL *vs* GDM analysis showed 70 proteins to be significantly different with S100 calcium-binding protein A9 (S100A9) the most different between the groups. (Table S1, FDR<10%). Similar analysis on CTRL vs PGD groups showed 77 proteins to be significantly different, with S100A9 as one of the 7 proteins reaching FDR<10% (Table S2).

We used MetaboAnalyst suite to visualize the proteomic data. Partial Least Squares Discriminant (PLSD) analysis (Figures 1A–1C) showed a clear separation between CTRL and GDM proteins early in pregnancy. To determine which proteins contribute to this discrimination we conducted the Variable Importance in Projection (VIP) analysis (Figure 1D). Table S3 shows that 107 proteins contributed to this discrimination between CTRL and GDM groups, with a VIP score of >1.5, and S100A9 topped this list with a VIP score of 7.7.A similar analysis between CTRL and PGD groups showed clear distinction between protein expressions in the 2 groups and S100A9 with the top VIP score of 4.09 (Table S4).

### S100A9 Protein Upregulation in Early Pregnancy Urine Exosome of Diabetic Subjects is Validated by Western Immunoblotting

We validated the statistically significant upregulation of S100A9 protein in diabetic urine exosomes by western immunoblotting analysis. Equal amounts (50 μg) of protein from each exosome sample was used, (Figure 2A, 2B) showing significant S100A9 upregulation in diabetic exosome. However, all diabetic patients did not uniformly show S100A9 upregulation. We therefore sought to further determine if the demographic feature differences between subjects of the same group correlate with the relative abundance of this protein as denoted by its peptide count.

### S100A9 Protein Peptide Count Data Correlates with Obesity and Macrosomia Differently in GDM *versus* PGD subjects

The average urinary exosome load of S100A9 peptide count for the PGD group was 22.25±37.89 (N=10) while the GDM had an average peptide count of 23.63±34.7 (N=8). Both were significantly different from the CTRL subject peptide count of 1.15±2.39 (N = 10) (p=0.0065, GDM *vs* CTRL and p=0.0174, PGD *vs* CTRL), which also closely tracked with the western blot data.

The average S100A9 peptide count was significantly different in the PGD and GDM subjects (7.4±13.43 *versus* 29.08±38.78, p=0.0209). When level of control of disease within the group was considered, PGD patients with well controlled disease have a lower average peptide count(0.25±0.5) compared with the poorly controlled group of PGD patients. It is noteworthy that both of these averages are less than the GDM group average.

GDM mothers with macrosomicneonates of had an average peptide count of 32.67±54.4 while GDM mothers of non-macrosomic neonates had an average peptide count of 25.5±17.85. Whereas this peptide count difference is likely less significant, it does illustrate a higher maternal peptide count for macrosomic neonates. Interestingly, a significantly higher rate of macrosomia in GDM than in PGD was observed (p=0.0002).

Lastly, we considered BMI. 6 of the 7 PGD patients and 4 of the 6 GDM patients were obese. Peptide count average of obese PGD patients was 6.25±12.44, while that of a single non-obese PGD patient was 0. In the GDM group, peptide average of obese patients was 42.5±37.53 and only 2.25±2.87 for non-obese patients. In the non-diabetic group, the peptide average of the obese subjects was 11.5±4.04 and only 1.67±1.51 for non-obese subjects. This strong trend (p=0.0585) demonstrates that peptide count is higher in pregnant obese women than in pregnant non-obese women.

### Urine Exosome Protein Content Changes from Early to Late Pregnancy Proceed *via* Different Pathways in CTRL *versus* PGD *versus* GDM

Further, we sought to examine how progression of pregnancy from an early time point to a later time point is reflected in the urine exosome proteins and pathways in these three groups. We performed a nested analysis on urine exosomes from 4 CTRL, 5 GDM and 3 PGD subjects that completed the 36^th^ week collection in addition to the 20^th^ week samples.

We observed considerable differences between exosome proteomes of early *versus* late pregnancy. For the 20^th^ and 36^th^ week samples respectively, 357 and 268 proteins (CTRL), 460 and 396 proteins (GDM) and 369 and 352 proteins (PGD) were identified. Thus the number of proteins identified in urine exosome generally decreased from early to late pregnancy. Groupwise analyses of the 20^th^ and 36^th^ week samples showed that the number of unique proteins identified reduced 5-fold in CTRL, roughly 2-fold in GDM and only slightly decreased in PGD groups (Figures S2A and S2B).

Thus, although the number of total pregnancy proteins identified was the highest in the GDM group (GDM>PGD>CTRL with 530,470 and 377 proteins respectively), within a group, the newer proteins identified during later pregnancy was CTRL < GDM <PGD with 20, 70 and 101 proteins respectively.

In addition, the protein pathways predominantly contributing to changes in progression of pregnancy from week 20 to week 36 were different in each group (Table S5, Figures S3A–S3C). We observed differences even in the extent to which a protein expression changed in one group *versus* the other (Δ_1_ =Protein1 NSAF_Week20_ – Proteinl NSAF_Week36_). Thus, Δ _1_ (for a given protein) for CTRL was different from Δ _1_ for GDM, which in turn was different from Δ _1_ for PGD group.

## Discussion

Urine comprises at least two distinct exosome populations: exosomes that are produced by pre-glomerular organs such as brain and muscle that are filtered from plasma into urine, and those that are produced by the kidney tubular cells distal to glomerular filtration. Urine exosome proteins represent only 3% of the whole urine proteome [23] and their cellular nature allows for the strong likelihood that this 3% contains cell- and cell-state specific markers derived from both renal and pre-renal organs. Given that every living surface is shown to use these bio-active nanoparticles for intercellular communication, [24] urine exosomes potentially have a systemic level representation, and their analysis provides clinically relevant information in describing a disease phenotype such as diabetes during pregnancy. Two recent exosome proteomics analysis from **(a)** Pisitkun, et al. show 295 distinct proteins at least 22 of which have been implicated in various kidney and systemic abnormalities [25] and **(b)** Zhou, et al. show the utility of exosomes as a source of kidney physiology biomarkers in AKI subjects [26].

In this report, we examined the difference in urine exosome protein content of diabetic and non-diabetic pregnancy subjects. In all, we found that 1103 proteins were identifiable from a total of 28 urine exosome samples of nondiabetic and diabetic subjects. Using three independent methods, namely negative binomial distribution for linear regression analysis, partial least squares discriminant analysis, and variable importance in projection analysis of the proteomic data, we have shown that the calcium binding protein S100 A9 is significantly upregulated in both pregestational as well as gestational diabetic pregnancies. S100A9 upregulation in diabetics was validated by western immunoblotting. We also showed that the peptide number of this protein independently correlates with macrosomia in the newborn infant as well as maternal obesity, although to a different extent in the GDM *versus* PGD groups. Though the mechanism of S100A9 upregulation in diabetes/obesity and its clinical significance needs to be established, other recent studies [27–29] have demonstrated S100A9 upregulation in diabetes or obesity, further supporting our finding.

S100A9 upregulation as a biomarker of inflammatory processes and immune response is gaining fast ground. S100A9 heterotetramerizes with S100A8 to form calprotectin,which has a plethora of intracellular and extracellular functions.It is predominantly localized in the cytoplasm but is translocated to the cytoskeleton and cell membrane upon elevation of intracellular calcium level, and gets secreted to extracellular space to amplify innate immune response by acting as an endogenous Danger-Associated Molecular Pattern (DAMP) that promotes inflammation [30]. To our knowledge, this report is the first study showing S100A9 elevation in the urinary exosomes of human subjects with obese/diabetic pregnancy. Given its ability to induce neutrophil chemotaxis and adhesion, a role for S100A9 in exosome vesicle trafficking cannot be ruled out. If so, it would be interesting to address whether S100A9 would play a role in increased exosome production in diabetic milieu.

This study has numerous strengths. The 24-hour urine sample collection design lends itself to reduced variation and is more representative of the phenotype. Collection of samples at two time points from the same individuals allows for quantification of intra-subject variability. Additionally, by virtue of their lipid lamellar structure, exosomes serve to transport hydrophobic proteins, so the analysis is not limited to hydrophilic proteins only. One limitation of this study is its small sample sizes. The S100A9 upregulation should be confirmed in a larger population, with subjects that are more tightly grouped together and matched with respect to age, gravidity, and ethnicity. Additionally, the pre-gestational diabetic group did not include any patients with Type 1 diabetes, and therefore the results of this study are limited in their application to Type 1 diabetics and further studies in this population are needed. In our PGD population the date of diabetic diagnosis, and therefore duration of their disease, was not collected. As a result we were unable to analyze the data with regards to duration of disease. Further, the PGD *vs* GDM difference in S100A9 peptide count also needs to be verified in a larger population. It appears that when the data within the PGD group peptide count is considered, higher peptide count is associated with poorer diabetes control. Possibly by extending the time points of sample collection to include the 4^th^-8^th^ week of pregnancy and 6 weeks after childbirth, this finding can be further validated. By developing a list of plausible risk-stratifiers, non-diabetic and/or non-obese pregnant women at risk for developing diabetes can potentially benefit from these studies.

Finally, we used 1d gel electrophoresis to resolve the exosome proteins prior to LC/MS-MS analysis, which resulted in identification of 1103 proteins overall. If we had conducted direct exosome protein trypsinization instead of following this method, perhaps the number of identified proteins might have increased. In our experience, exosomes cargo contains non-full length peptides as well, and has the potential to confound the analysis. By following the gel electrophoresis method, we ensured that we only compare full length protein differences between groups.

## Conclusion

In this study, we summarize our initial findings from diabetic pregnancy urine exosome proteomic analyses. We used exosomes from 24 hour urine samples of diabetic pregnancy subjects obtained during the 20^th^ week of pregnancy and compared them with exosomes from nondiabetic pregnancy subjects. Our data show major alteration to calcium-handling pathways and cellular-danger signal pathways between groups. The major finding of this study is that the DAMP protein S100A9 sorts into exosomes, and in diabetics this exosomal sorting is upregulated. The exosomal load of this protein correlates with not only maternal obesity but also macrosomia of the new born infant. Further, developmental changes from week 20 to week 36 in GDM and PGD phenotypes employ different protein pathways.

Given that maternal diabetes during human pregnancy alters the quality of life of both the mother and the newborn after childbirth, and considering the non-invasive nature of urinary exosome collection, processing and analysis of 24 hour urine samples is an attractive platform to study diabetes during pregnancy and its consequences.

Work from the Knepper group shows that many important renal proteins (e.g. aquaporins, polycystins and podocyn) are shed in the urine exosome. [25; 31] Our current report adds S100A9 to this group of functionally important proteins to be identified in urine exosomes. Our findings, together with other studies in pancreatic cancer tissue, saliva, and serum suggest that the upregulation of S100A9 DAMP signal is a common and valid biomarker of inflammatory processes and immune response. This report potentially forms the preliminary level basis for future studies wherein the pharmacologic and therapeutic potential of S100A9 and related pathways could be utilized to impart personalized care to a pregnant woman with diabetes.

## Supplementary Material

1

## Figures and Tables

**Figure 1A:**
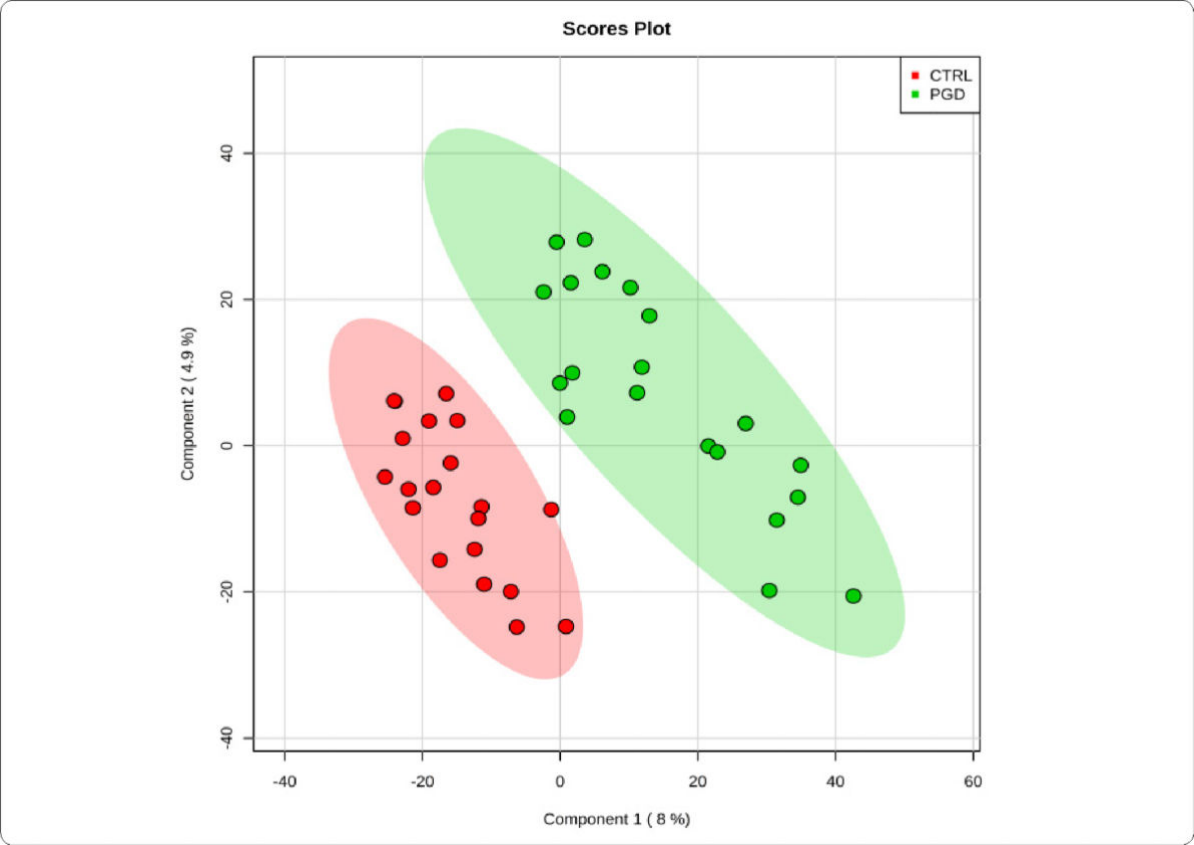
PGD *vs.* controlconcentration-based proteomic measurements: PLSDA separation using protein NSAF measurements in the urine exosome of Pre-Gestational Diabetes Mellitus (PGD,n = 10) vs Control subjects (CTRL,n = 10). Lack of overlap between the two groups of exosome proteins signifies clear separation of PGD from CTRL.

**Figure 1B:**
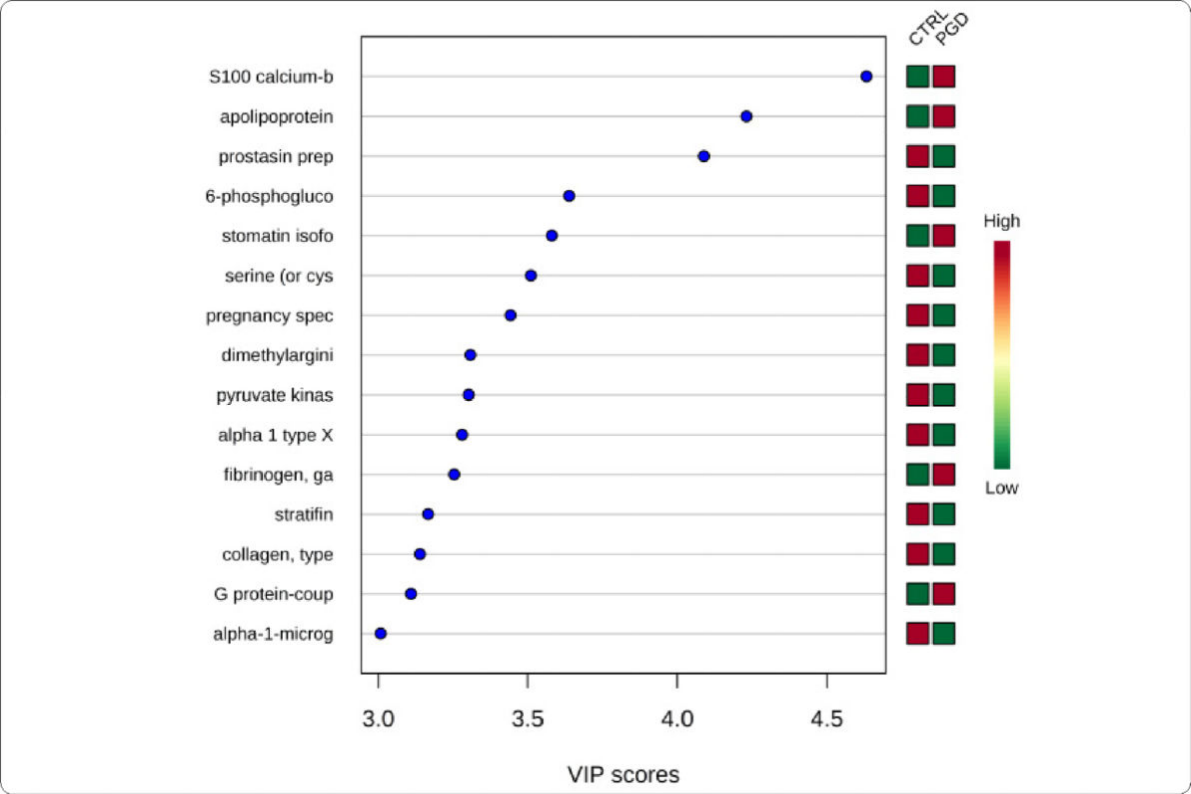
PGD *vs.* Control variable importance in projection plot: Urinary exosome proteins identified by PLS-DA in a descending order of importance. The graph represents relative contribution of proteins to the variance between the PGD and CTRL urine exosomes. The green and red boxes on the right indicate whether the protein concentration is increased (red) or decreased (green) in the exosome of the PGD urine vs. CTRL urine samples. Similar to analysis in Figure 1b, S100A9 is the top protein (VIP score of >4.5).

**Figure 1C:**
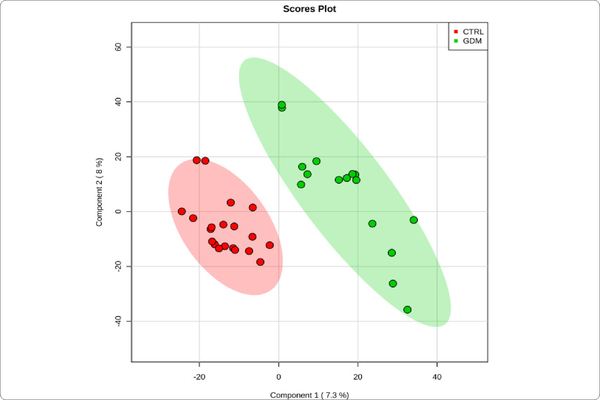
GDM *vs.* control concentration-based proteomic measurements: Two-dimensional (2D) partial least squares discriminant analysis (PLSDA) separation using protein normalized spectral abundancy factor (NSAF) concentration-based proteomic measurements in the urine exosome of Gestational Diabetes Mellitus (GDM, n=8) vs Control subjects (CTRL, n = 10). Clear separation of GDM from CTRL is observed.

**Figure 1D:**
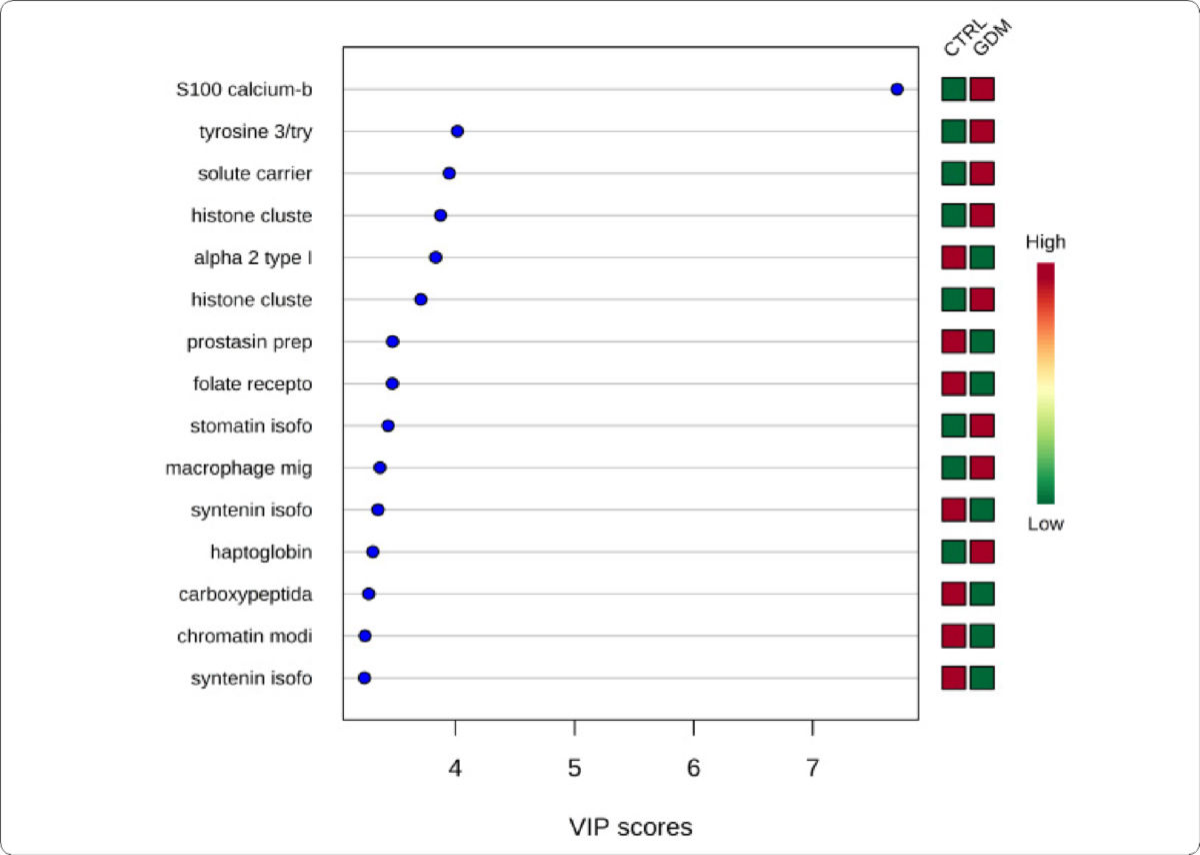
GDM vs. control variable Importance In projection plot: The graph represents relative contribution of proteins to the variance between the GDM and CTRL urine exosomes at week 20 of pregnancy. High value of VIP score for a protein indicates greater contribution of the protein to the separation of groups. The green and red boxes on the right indicate whether the protein concentration is increased (red) or decreased (green) in the exosome of the GDM urine vs. CTRL urine samples. For higher n value, a VIP score of 1.5 is considered to enable discrimination between 2 phenotypes. Even with the low n (=10) per group that is employed in this study, the VIP score of the top 3 proteins is higher than 3. S100 calcium binding protein A9 is the top protein with a VIP score of >7.

**Figure 2A:**
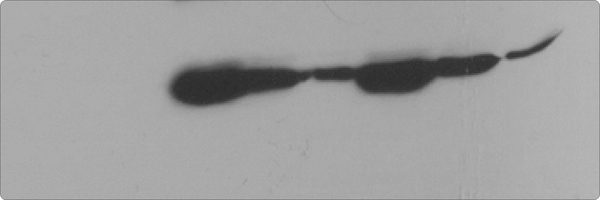
Immunoblotting of pregnancy urine exosome for S100A9 protein. Lanes 1–3: control; lanes 4–6: GDM; lanes 7–9: PGD.

**Figure 2B:**
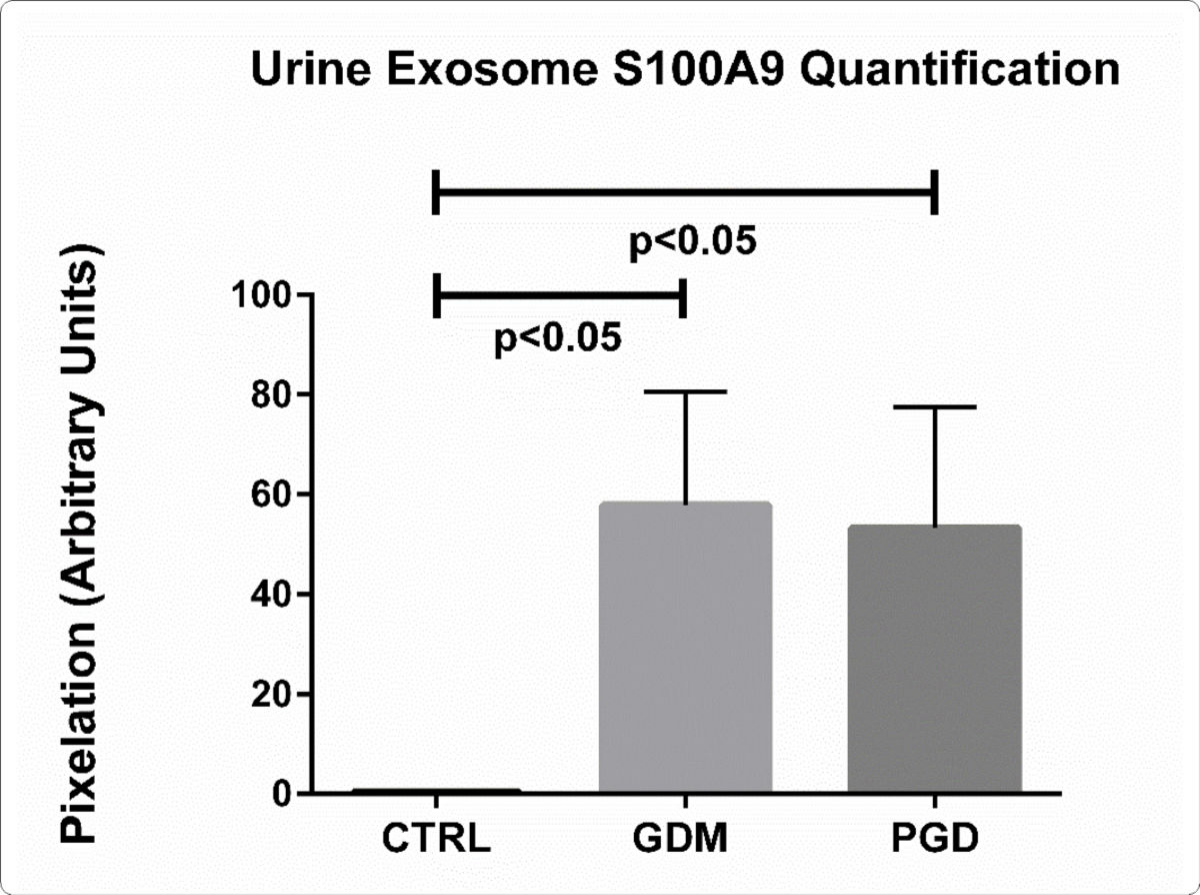
Quantification of S100A9 from immunoblots in A (control, n=3; GDM, n=3, PGD, n=3). Data are means ± SEM. p<0.05 pregnancy urine exosome S100A9 GDM *versus* CTRL; PGD *versus* CTRL.

**Table 1:** Patient Demographics.

Variable	PGD, N=7	GDM, N=6	Control, N=6	P value

Age	30.9±8.1	30.7±7.1	33.2±5.8	0.84

Race/Ethnicity				
White	0	0	3	0.04
Black	2	1	0	0.76
Asian	0	1	0	0.56
Hispanic	4	4	2	0.13
Other	2	0	1	0.21

Weight (kg)	87±17.2	89.1±20.4	75.6±13.4	0.48

BMI (m/kg^2^)	33.9±4.3	33.6±6.6	27.6±6.8	0.2

HgBAIC (%) First Trimester	9.2±1.7	6±0.28	5.3±0.17	0.0003
Second Trimester	7.3±1.2	5.9±0.35	NA	0.09
Third Trimester	6.7±0.8	6	NA	0.48

Fasting Plasma Glucose (mg/dL)	92.4±18.3	92±18.3	74±12.5	0.3

75 mg 2 Hour Oral Glucose Tolerance Test Glucose Level (mg/dL)	NA	170.5±79.9	85.5±21.9	NA

Medications				
Oral	2	2	0	0.13
Insulin	6	0	0	0.13

Pre-eclampsia	1	0	0	0.04

Gestational Hypertension	2	0	0	0.47

Retinopathy	0	0	0	NA

Chronic Hypertension	3	0	0	0.2

Nephropathy	0	0	0	NA

**Table 2:** Neonatal Outcomes.

Variable	PGD, N=7	GDM, N=6	Control, N=6	P value
Gestational Age (weeks, days)	37 weeks 5 days	38 weeks 3 days	38 weeks 2 days	0.863
Birth Weight (grams)	3155±697	3555±232	3173±285	0.666
Birth Length (cm)	50.1±3	51.1±2.7	49.6±1.6	0.672
Head circumference (cm)	33.6±2.3	34.5±1	34±1.4	0.729

## Abbreviations

PGD: Pre-gestational diabetic
MVBs: Multivesicular bodies
PLS-DA: Partial-Least Squares Discriminant Analysis
VIP: Variable Importance in Projection
FDR: False Discovery Rate
PLSD: Partial Least Squares Discriminant
DAMP: Danger-Associated Molecular Pattern
