# Supplementary material for: Proteomic analyses of Urine Exosomes reveal New Biomarkers of Diabetes in Pregnancy
Source: Madridge J Diabetes. Author manuscript; Available in PMC 2019 Aug 23. (PMC6707737; doi:10.18689/mjd-1000103)
Supplement: 1 [file NIHMS1028115-supplement-1.pdf]

Supplementary Data

Sl. #	GI number	p-val	p-val FDR	Protein Identification
1	4506773	0.00003	0.03686	S100 calcium-binding protein A9
2	4502085	0.00074	0.23121	pancreatic amylase alpha 2A precursor
3	20070125	0.00091	0.23121	prolyl 4-hydroxylase, beta subunit precursor
4	21614544	0.00106	0.23121	S100 calcium-binding protein A8
5	77539758	0.00111	0.23121	histone cluster 2, H4b
6	24497480	0.00126	0.23121	solute carrier family 22 member 6 isoform d
7	4504299	0.00193	0.26478	histone cluster 3, H3
8	5803023	0.00222	0.26478	lectin, mannose-binding 2
9	55956899	0.00244	0.26478	keratin 9
10	13162290	0.00262	0.26478	betaine-homocysteine methyltransferase 2
11	21361176	0.00282	0.26478	aldehyde dehydrogenase 1A1
12	119964718	0.00316	0.26478	desmoglein 3 preproprotein
13	38016911	0.00321	0.26478	stomatin isoform a
14	21361392	0.00338	0.26478	pregnancy specific beta-1-glycoprotein 1
15	4506153	0.0036	0.26478	prostasin preproprotein
16	4507033	0.00423	0.28534	solute carrier family 5 (sodium/glucose cotransporter), member 2
17	5031839	0.0044	0.28534	keratin 6A
18	4809279	0.00518	0.28923	annexin VII isoform 2
19	4826762	0.00521	0.28923	Haptoglobin
20	66912162	0.00542	0.28923	histone cluster 2, H2bf
21	31652249	0.00551	0.28923	lipopolysaccharide-binding protein precursor
22	9257217	0.0065	0.32589	folate receptor 1 precursor
23	119395754	0.00718	0.34409	keratin 5
24	44680145	0.00795	0.36555	solute carrier family 23 (nucleobase transporters), member 1 isoform a
25	4557317	0.00871	0.38447	annexin A11
26	37694062	0.0105	0.42637	aquaporin 1
27	4503029	0.01078	0.42637	cellular retinoic acid binding protein 2
28	4557581	0.01082	0.42637	fatty acid binding protein 5 (psoriasis-associated)
29	4502211	0.01197	0.45516	ADP-ribosylation factor 6
30	12408656	0.01304	0.45735	calpain 1, large subunit
31	157805480	0.01339	0.45735	pregnancy specific beta-1-glycoprotein 7 precursor
32	32401453	0.01385	0.45735	solute carrier family 36 (proton/amino acid symporter), member 2
33	153945728	0.01387	0.45735	microtubule-associated protein 1B
34	4757944	0.01417	0.45735	CD81 antigen
35	32189392	0.01474	0.45735	peroxiredoxin 2 isoform a
36	98986445	0.01493	0.45735	carcinoembryonic antigen-related cell adhesion molecule 5 preproprotein
37	48762934	0.01677	0.49797	alpha 2 type I collagen
38	21614513	0.01749	0.01749	aldehyde dehydrogenase 1 family, member L1
39	4557367	0.01767	0.49797	bleomycin hydrolase
40	51468073	0.01806	0.49797	solute carrier family 6, member 19
41	4504517	0.02	0.51727	heat shock 27kDa protein 1
42	54873613	0.02026	0.51727	Agrin
43	109255249	0.02141	0.51727	keratin 4
44	50592994	0.02152	0.51727	Thioredoxin
45	116174789	0.02264	0.51727	centrosome spindle pole associated protein 1 isoform a
46	157671931	0.02268	0.51727	solute carrier family 5 (sodium/glucose cotransporter), member 12
47	87159824	0.0231	0.51727	CD151 antigen
48	116008152	0.02352	0.51727	alpha 1 type XV collagen precursor
49	134254459	0.02413	0.51727	sodium potassium chloride cotransporter 2
50	126012573	0.02441	0.51727	low density lipoprotein-related protein 2
51	116256354	0.02458	0.51727	alpha 2 type IV collagen preproprotein
52	4504349	0.02481	0.51727	beta globin
53	4504763	0.02486	0.51727	integrin alpha-V precursor
54	59850812	0.02623	0.53575	uromodulin precursor
55	7656922	0.02705	0.54245	chromatin modifying protein 2A
56	4758236	0.02946	0.5802	extracellular matrix protein 1 isoform 1 precursor
57	7019485	0.03005	0.03005	programmed cell death 6
58	18765694	0.03076	0.58264	dipeptidylpeptidase IV
59	134244281	0.03117	0.58264	melanoma-associated antigen p97 isoform 1, precursor
60	14150145	0.03211	0.59037	matrix-remodelling associated 8
61	28603818	0.03476	0.62852	hypothetical protein LOC338094
62	29788785	0.03733	0.65087	tubulin, beta
63	119964726	0.03744	0.65087	insulin-like growth factor 2 receptor precursor
64	21361254	0.03777	0.65087	deoxyribonuclease I precursor
65	4502101	0.03859	0.65487	annexin I
66	4503899	0.04423	0.73915	N-acetylgalactosamine-6-sulfatase precursor
67	39995109	0.04539	0.74722	GM2 ganglioside activator precursor
68	87239981	0.0475	0.76713	tankyrase, TRF1-interacting ankyrin-related ADP-ribose polymerase
69	4826898	0.76713	0.76713	profilin 1
70	42741659	0.04951	0.77688	ATP-binding cassette sub-family B member 1

Table S1: Significantly dysregulated proteins in the GDM group by negative binomial distribution analysis

Sl. #	GI number	p-val	p-val FDR	Protein Identification
1	4502027	0.00006	0.02631	albumin precursor
2	38016911	0.00008	0.02631	stomatin isoform a
3	4506773	0.00009	0.02631	S100 calcium-binding protein A9
4	31652249	0.0001	0.02631	lipopolysaccharide-binding protein precursor
5	122937470	0.00021	0.04555	carboxypeptidase N, polypeptide 2, 83kD
6	115298678	0.00025	0.04555	complement component 3 precursor
7	4504811	0.00061	0.09676	junction plakoglobin
8	4506153	0.00104	0.1336	prostasin preproprotein
9	21264363	0.00109	0.1336	mannan-binding lectin serine protease 2 isoform 1 precursor
10	86793109	0.00156	0.16728	complement receptor 1 isoform S precursor

11	6912586	0.00169	0.16728	6-phosphogluconolactonase
12	58530840	0.00182	0.16728	desmoplakin isoform I
13	65506891	0.00216	0.17759	solute carrier family 3 (activators of dibasic and neutral amino acid transport), member 2 isoform c
14	4503491	0.00225	0.17759	epidermal growth factor (beta-urogastrone)
15	126012573	0.00294	0.20392	low density lipoprotein-related protein 2
16	4502179	0.00319	0.20392	aquaporin 2
17	24497480	0.00325	0.20392	solute carrier family 22 member 6 isoform d
18	115298674	0.00345	0.20392	nidogen 1 precursor
19	21361181	0.00351	0.20392	Na+/K+ -ATPase alpha 1 subunit isoform a proprotein
20	126091152	0.0037	0.20392	cubilin
21	6382064	0.0042	0.21566	0.21566
22	154146262	0.0044	0.21566	Fc fragment of IgG binding protein
23	116256354	0.00456	0.21566	alpha 2 type IV collagen preproprotein
24	7524354	0.00469	0.21566	dimethylarginine dimethylaminohydrolase 2
25	73858570	0.00498	0.21982	complement component 1 inhibitor precursor
26	116008152	0.00579	0.23976	alpha 1 type XV collagen precursor
27	4502067	0.00604	0.23976	alpha-1-microglobulin/bikunin precursor
28	4503635	0.00609	0.23976	coagulation factor II precursor
29	31542984	0.00632	0.24053	inter-alpha (globulin) inhibitor H4
30	4504965	0.00765	0.28112	L-plastin
31	18765694	0.00829	0.29513	dipeptidylpeptidase IV
32	37694062	0.00944	0.31977	aquaporin 1
33	4758950	0.00957	0.31977	peptidylprolyl isomerase B precursor
34	153945728	0.01071	0.34747	microtubule-associated protein 1B
35	88853069	0.01115	0.35145	vitronectin precursor
36	48762934	0.01237	0.37128	alpha 2 type I collagen
37	51468073	0.01279	0.37128	solute carrier family 6, member 19
38	4502511	0.01284	0.37128	complement component 9
39	27894376	0.01313	0.37128	cell adhesion molecule with homology to L1CAM precursor
40	21264578	0.01375	0.37905	tetraspan 1
41	126012571	0.0148	0.3981	heparan sulfate proteoglycan 2
42	4506403	0.01587	0.40814	G protein-coupled receptor, family C, group 5, member A
43	11321561	0.01591	0.40814	hemopexin
44	7706451	0.01685	0.42238	G protein-coupled receptor, family C, group 5, member B precursor
45	58761548	0.01747	0.42812	tau tubulin kinase 1
46	21071030	0.01872	0.44894	alpha 1B-glycoprotein precursor
47	4507033	0.02107	0.48572	solute carrier family 5 (sodium/glucose cotransporter), member 2
48	91105159	0.02114	0.48572	CD14 antigen precursor
49	4502503	0.02162	0.48677	complement component 4 binding protein, alpha chain precursor
50	10863877	0.02247	0.48742	phospholipid scramblase 1
51	59850812	0.02297	0.48742	uromodulin precursor
52	4507377	0.02298	0.48742	serine (or cysteine) proteinase inhibitor, clade A, member 7
53	134254459	0.02377	0.49073	sodium potassium chloride cotransporter 2
54	116256333	0.02414	0.49073	membrane metallo-endopeptidase
55	7706635	0.02461	0.49073	cornulin
56	134244281	0.02491	0.49073	melanoma-associated antigen p97 isoform 1, precursor
57	124256496	0.0268	0.51852	heat shock 70kDa protein 1-like
58	53729346	0.02772	0.5246	plakophilin 1 isoform 1b
59	4503689	0.02806	0.5246	fibrinogen, alpha polypeptide isoform alpha-E preproprotein
60	4502085	0.0292	0.53519	pancreatic amylase alpha 2A precursor
61	21361176	0.0296	0.53519	aldehyde dehydrogenase 1A1
62	4502105	0.03044	0.54091	annexin IV
63	4557759	0.0309	0.54091	myeloperoxidase
64	4503549	0.03192	0.55011	elastase 2, neutrophil preproprotein
65	40217833	0.03353	0.55862	G protein-coupled receptor family C, group 5, member C isoform b
66	87196339	0.03359	0.55862	collagen, type VI, alpha 1 precursor
67	49574489	0.03434	0.55862	Na+/K+ -ATPase beta 1 subunit isoform b
68	4504763	0.03482	0.55862	integrin alpha-V precursor
69	112380628	0.03495	0.55862	lysosomal-associated membrane protein 1
70	5031857	0.03669	0.57818	lactate dehydrogenase A
71	5123454	0.03775	0.58646	heat shock 70kDa protein 1A
72	45439327	0.04147	0.04147	periplakin
73	109255249	0.04235	0.63491	keratin 4
74	4503143	0.0426	0.63491	cathepsin D preproprotein
75	4504061	0.04344	0.63892	glucosamine (N-acetyl)-6-sulfatase precursor
76	5174387	0.04407	0.63957	prominin 1
77	9665262	0.04894	0.7011	EGF-containing fibulin-like extracellular matrix protein 1 precursor

Table S2: Significantly dysregulated proteins in the PGD group by negative binomial distribution analysis

Sl. #	GI #	VIP scores	Annotations
1	4506773	7.7075	S100 calcium-binding protein A9
2	5803227	4.0166	tyrosine 3/tryptophan 5 -monooxygenase activation protein, theta polypeptide
3	65506891	3.9476	solute carrier family 3 (activators of dibasic and neutral amino acid transport), member 2 isoform c
4	4504299	3.8755	histone cluster 3, H3
5	116256354	3.8346	alpha 2 type IV collagen preproprotein
6	77539758	3.709	histone cluster 2, H4b
7	4506153	3.4717	prostasin preproprotein
8	9257217	3.4696	folate receptor 1 precursor
9	38016911	3.434	stomatin isoform a
10	4505185	3.3661	macrophage migration inhibitory factor (glycosylation-inhibiting factor)
11	56243522	3.3497	syntenin isoform 1
12	4826762	3.3065	haptoglobin
13	122937470	3.2718	carboxypeptidase N, polypeptide 2, 83kD
14	7656922	3.2405	chromatin modifying protein 2A
15	55749523	3.2358	syntenin isoform 3
16	77539055	3.2276	ubiquitin and ribosomal protein L40 precursor
17	31652249	3.214	lipopolysaccharide-binding protein precursor
18	4809279	3.0934	annexin VII isoform 2
19	32189392	2.9747	peroxiredoxin 2 isoform a
20	21361181	2.948	Na+/K+ -ATPase alpha 1 subunit isoform a proprotein
21	24234699	2.9417	keratin 19
22	4504253	2.8854	H2A histone family, member X
23	21614544	2.8484	S100 calcium-binding protein A8
24	4503117	2.8454	cystatin B
25	66912162	2.7888	histone cluster 2, H2bf
26	4826898	2.7639	profilin 1
27	116008152	2.6908	alpha 1 type XV collagen precursor
28	4502211	2.6618	ADP-ribosylation factor 6
29	7019485	2.6024	programmed cell death 6
30	9966777	2.5502	resistin
31	5031857	2.5453	lactate dehydrogenase A
32	4557287	2.536	angiotensinogen preproprotein
33	21361176	2.4817	aldehyde dehydrogenase 1A1
34	7705885	2.4528	vacuolar protein sorting 28 isoform 1
35	4504349	2.4504	beta globin
36	40354205	2.4308	aldolase B
37	14150145	2.402	matrix-remodelling associated 8
38	20070125	2.3701	prolyl 4-hydroxylase, beta subunit precursor
39	155969697	2.3701	keratin 6C
40	164663836	2.3322	pregnancy specific beta-1-glycoprotein 11 isoform 1
41	119964718	2.2922	desmoglein 3 preproprotein
42	11386147	2.2884	prosaposin isoform a preproprotein
43	4504347	2.2462	alpha 1 globin
44	17921989	2.1848	tubulin, alpha 4a
45	24308440	2.1839	family with sequence similarity 125, member A
46	4885179	2.1769	defensin, alpha 3 preproprotein

47	157671931	2.1546	solute carrier family 5 (sodium/glucose cotransporter), member 12
48	37694062	2.1335	aquaporin 1
49	19913424	2.1262	ATPase, H+ transporting, lysosomal 70kD, V1 subunit A, isoform 1
50	4503029	2.1207	cellular retinoic acid binding protein 2
51	6598323	2.1154	GDP dissociation inhibitor 2
52	4885607	2.0963	small proline-rich protein 3
53	67191208	2.0877	ubiquitin C
54	89357932	2.0783	keratin 5b
55	50592994	2.0491	thioredoxin
56	4758984	2.0242	Ras-related protein Rab-11A
57	5803225	2.0225	tyrosine 3/tryptophan 5 -monooxygenase activation protein, epsilon polypeptide
58	13162290	2.0047	betaine-homocysteine methyltransferase 2
59	4757944	2.0019	CD81 antigen
60	6382064	1.9736	prostatic acid phosphatase precursor
61	21361392	1.957	pregnancy specific beta-1-glycoprotein 1
62	24497480	1.9544	solute carrier family 22 member 6 isoform d
63	32189394	1.9416	ATP synthase, H+ transporting, mitochondrial F1 complex, beta subunit precursor
64	4502101	1.939	annexin I
65	88853069	1.9077	vitronectin precursor
66	4503471	1.8901	eukaryotic translation elongation factor 1 alpha 1
67	24430144	1.8457	glutathione S-transferase A3
68	32401453	1.8451	solute carrier family 36 (proton/amino acid symporter), member 2
69	4557581	1.8396	fatty acid binding protein 5 (psoriasis-associated)
70	118582275	1.8343	superoxide dismutase 3, extracellular precursor
71	119703753	1.8246	1.8246
72	18765694	1.8066	dipeptidylpeptidase IV
73	12667788	1.7874	myosin, heavy polypeptide 9, non-muscle
74	5031809	1.7777	immunoglobulin superfamily containing leucine-rich repeat
75	49574489	1.7717	Na+/K+ -ATPase beta 1 subunit isoform b
76	8393956	1.7516	serine (or cysteine) proteinase inhibitor, clade B (ovalbumin), member 13
77	4507033	1.7399	solute carrier family 5 (sodium/glucose cotransporter), member 2
78	10280622	1.7383	amylase, pancreatic, alpha-2B precursor
79	4507377	1.7349	serine (or cysteine) proteinase inhibitor, clade A, member 7
80	4506979	1.7337	solute carrier family 13 (sodium-dependent dicarboxylate transporter), member 2
81	47419930	1.7058	chondroitin sulfate proteoglycan 4
82	44680145	1.6934	solute carrier family 23 (nucleobase transporters), member 1 isoform a
83	115298674	1.6917	nidogen 1 precursor
84	40068518	1.6912	phosphogluconate dehydrogenase
85	87159824	1.691	CD151 antigen
86	4885111	1.6836	calmodulin-like 3
87	4758236	1.6805	extracellular matrix protein 1 isoform 1 precursor
88	4557321	1.6767	apolipoprotein A-I preproprotein
89	4504517	1.6746	heat shock 27kDa protein 1
90	6912582	1.6657	penta-EF-hand domain containing 1
91	30089930	1.6653	N-acylsphingosine amidohydrolase (acid ceramidase) 1 isoform b
92	4502067	1.6635	alpha-1-microglobulin/bikunin precursor
93	17865802	1.6598	vacuolar protein sorting factor 4B
94	7524354	1.6502	dimethylarginine dimethylaminohydrolase 2
95	5031635	1.6369	cofilin 1 (non-muscle)
96	5803185	1.6322	synaptophysin-like 1 isoform a
97	20357529	1.6111	guanine nucleotide-binding protein, beta-2 subunit
98	4507025	1.5925	solute carrier family 4, sodium bicarbonate cotransporter, member 4 isoform 2
99	31542306	1.5884	chromatin modifying protein 1B
100	87196339	1.586	collagen, type VI, alpha 1 precursor
101	148536848	1.5748	carboxyl ester lipase precursor
102	23943854	1.5601	pepsinogen 5, group I
103	98986445	1.5597	carcinoembryonic antigen-related cell adhesion molecule 5 preproprotein
104	12408656	1.5565	calpain 1, large subunit
105	63252913	1.5509	gelsolin-like capping protein
106	29788785	1.5331	ubulin, beta
107	4506403	1.5005	G protein-coupled receptor, family C, group 5, member A

Table S3: Variable Importance in Projection Scores for proteins by spectral count, NL vs GDM

Sl. #	GI #	VIP scores	Annotations
1	4506773	4.632	S100 calcium-binding protein A9
2	4557321	4.2308	apolipoprotein A-I preproprotein
3	4506153	4.0883	prostasin preproprotein
4	6912586	3.6375	6-phosphogluconolactonase
5	38016911	3.5797	stomatin isoform a
6	4507377	3.5105	serine (or cysteine) proteinase inhibitor, clade A, member 7
7	164663836	3.4421	pregnancy specific beta-1-glycoprotein 11 isoform 1
8	7524354	3.3078	dimethylarginine dimethylaminohydrolase 2
9	33286418	3.302	pyruvate kinase, muscle isoform 1
10	116008152	3.2799	alpha 1 type XV collagen precursor
11	70906439	3.2533	fibrinogen, gamma chain isoform gamma-B precursor
12	5454052	3.1659	stratifin
13	87196339	3.1386	collagen, type VI, alpha 1 precursor
14	40217833	3.1087	G protein-coupled receptor family C, group 5, member C isoform b
15	4502067	3.0079	alpha-1-microglobulin/bikunin precursor
16	4757944	3.0035	CD81 antigen
17	5802984	2.9308	UDP-GlcNAc:betaGal beta-1,3-N-acetylglucosaminyltransferase 1
18	4504811	2.9082	junction plakoglobin
19	122937470	2.8826	carboxypeptidase N, polypeptide 2, 83kD
20	21361181	2.8526	Na+/K+ -ATPase alpha 1 subunit isoform a proprotein
21	73858570	2.8491	complement component 1 inhibitor precursor
22	115298674	2.7204	nidogen 1 precursor
23	10863877	2.6684	phospholipid scramblase 1
24	5123454	2.6459	heat shock 70kDa protein 1A
25	116256354	2.6122	alpha 2 type IV collagen preproprotein
26	18765694	2.5936	dipeptidylpeptidase IV
27	4557871	2.5471	transferrin
28	7706451	2.5324	G protein-coupled receptor, family C, group 5, member B precursor
29	24497480	2.4617	solute carrier family 22 member 6 isoform d
30	31377727	2.4543	solute carrier family 44, member 2
31	42716297	2.4472	clusterin isoform 1
32	4502179	2.4194	aquaporin 2
33	115298678	2.4178	complement component 3 precursor
34	31652249	2.4169	lipopolysaccharide-binding protein precursor
35	7706635	2.3959	cornulin
36	71773110	2.3568	apolipoprotein A-IV precursor
37	6382064	2.3309	prostatic acid phosphatase precursor
38	58530840	2.2471	desmoplakin isoform I
39	21071030	2.2143	alpha 1B-glycoprotein precursor
40	16418467	2.2057	leucine-rich alpha-2-glycoprotein 1
41	157671931	2.1969	solute carrier family 5 (sodium/glucose cotransporter), member 12
42	4507953	2.1733	tyrosine 3/tryptophan 5 -monooxygenase activation protein, zeta polypeptide
43	4503635	2.1671	coagulation factor II precursor
44	4505881	2.164	plasminogen
45	4826772	2.1637	insulin-like growth factor binding protein, acid labile subunit
46	21361392	2.1522	pregnancy specific beta-1-glycoprotein 1
47	4758950	2.1491	peptidylprolyl isomerase B precursor

48	24430192	2.1243	keratin 16
49	50659080	2.1066	serpin peptidase inhibitor, clade A, member 3 precursor
50	67782351	2.0669	complement component 4B preproprotein
51	4505591	2.0643	peroxiredoxin 1
52	4502205	2.0557	ADP-ribosylation factor 4
53	9257232	2.055	orosomucoid 1 precursor
54	13929462	2.0409	UDP-Gal:betaGlcNAc beta 1,4- galactosyltransferase 1, membrane-bound form
55	5174387	2.0403	prominin 1
56	prominin 1	2.0343	vacuolar protein sorting 37D
57	19913424	2.0339	ATPase, H+ transporting, lysosomal 70kD, V1 subunit A, isoform 1
58	21264363	2.0322	mannan-binding lectin serine protease 2 isoform 1 precursor
59	21361302	2.0251	serine (or cysteine) proteinase inhibitor, clade A (alpha-1 antiproteinase, antitrypsin), member 4
60	42740907	2.0186	clusterin isoform 2
61	21361176	1.9999	aldehyde dehydrogenase 1A1
62	34335272	1.9927	endothelial protein C receptor precursor
63	65506891	1.9858	solute carrier family 3 (activators of dibasic and neutral amino acid transport), member 2 isoform c
64	89903012	1.9843	cell division cycle 42 isoform 1
65	70906435	1.9481	fibrinogen, beta chain preproprotein
66	23943854	1.932	pepsinogen 5, group I
67	28395033	1.9184	ras homolog gene family, member C precursor
68	145701025	1.9094	multiple EGF-like-domains 8
69	5803023	1.9071	lectin, mannose-binding 2
70	61835217	1.8962	3-mercaptopyruvate sulfurtransferase
71	4504965	1.8932	L-plastin
72	16418397	1.8861	MAL2 proteolipid protein
73	4503491	1.8649	epidermal growth factor (beta-urogastrone)
74	4506403	1.8364	G protein-coupled receptor, family C, group 5, member A
75	4502403	1.8286	biglycan preproprotein
76	21389623	1.8208	prominin 2
77	51468073	1.8142	solute carrier family 6, member 19
78	7661678	1.8103	RAP1B, member of RAS oncogene family
79	5803185	1.7818	synaptophysin-like 1 isoform a
80	119703753	1.7729	keratin 6B
81	24111250	1.7602	guanine nucleotide binding protein (G protein), alpha 13
82	4502085	1.7546	pancreatic amylase alpha 2A precursor
83	5902072	1.7323	serine (or cysteine) proteinase inhibitor, clade B (ovalbumin), member 3
84	4502511	1.719	complement component 9
85	91105159	1.7171	CD14 antigen precursor
86	42761474	1.6983	CD59 antigen p18-20
87	40217831	1.6803	G protein-coupled receptor family C, group 5, member C isoform a
88	37694062	1.6679	aquaporin 1
89	148612887	1.6665	choline transporter-like protein 4
90	4503143	1.6648	cathepsin D preproprotein
91	31542984	1.661	inter-alpha (globulin) inhibitor H4
92	12667788	1.6575	myosin, heavy polypeptide 9, non-muscle
93	11321561	1.642	hemopexin
94	6912236	1.6322	angiopoietin-like 2 precursor
95	89357932	1.6119	keratin 5b
96	21361195	1.5958	serine (or cysteine) proteinase inhibitor, clade A (alpha-1 antiproteinase, antitrypsin), member 5
97	41152114	1.5949	aldo-keto reductase family 7, member A3
98	24308440	1.5879	family with sequence similarity 125, member A
99	4507033	1.5843	solute carrier family 5 (sodium/glucose cotransporter), member 2
100	63252913	1.5821	gelsolin-like capping protein
101	67190748	1.5632	complement component 4A preproprotein
102	4502599	1.5364	carbonyl reductase 1
103	73858564	1.5172	corticosteroid binding globulin precursor

Table S4: Variable Importance in Projection Scores for proteins by spectral count, NL vs PGD

GI #	VIP Scores	Top discriminating Proteins in the CTRL group
4885049	8.093	cardiac muscle alpha actin 1 proprotein
4501889	6.1709	actin, gamma 2 propeptide
42716297	5.9226	clusterin isoform 1
42740907	4.6241	clusterin isoform 2
14389309	3.7985	tubulin alpha 6
67782351	3.4398	complement component 4B preproprotein
153792590	3.2261	heat shock protein 90kDa alpha (cytosolic), class A member 1 isoform 1
33286422	3.1219	pyruvate kinase, muscle isoform 2
19923483	3.0449	GTPase Rab14
33695095	2.967	ras-related GTP-binding protein RAB10
**GI #**	**VIP Scores**	**Top discriminating Proteins in the GDM group**
4503143	4.0378	cathepsin D preproprotein
17986283	3.9943	tubulin, alpha 1a
4502317	3.8459	vacuolar H+ ATPase E1 isoform a
7706683	3.5649	Rh family, C glycoprotein
19913428	3.5516	vacuolar H+ATPase B2
4885049	3.5127	cardiac muscle alpha actin 1 proprotein
14389309	3.4618	tubulin alpha 6
21361181	3.4455	Na+/K+ -ATPase alpha 1 subunit isoform a proprotein
19913426	3.4341	ATPase, H+ transporting, lysosomal 56/58kDa, V1 subunit B1
11545731	3.3769	gigaxonin
**GI #**	**VIP Scores**	**Top discriminating Proteins in the PGD group**
21361181	4.1922	Na+/K+ -ATPase alpha 1 subunit isoform a proprotein
4885049	4.136	cardiac muscle alpha actin 1 proprotein
169171979	3.9129	PREDICTED: similar to hCG1811191
4502111	3.7935	annexin VII isoform 1
7706683	3.7318	Rh family, C glycoprotein
42741659	3.636	ATP-binding cassette sub-family B member 1
7706451	3.5819	G protein-coupled receptor, family C, group 5, member B precursor
31377727	3.4821	solute carrier family 44, member 2
21389623	3.3071	prominin 2
42740907	3.1718	clusterin isoform 2

Table S5: CTRL, GDM and PGD VIP protein differences between week 20 and week 36

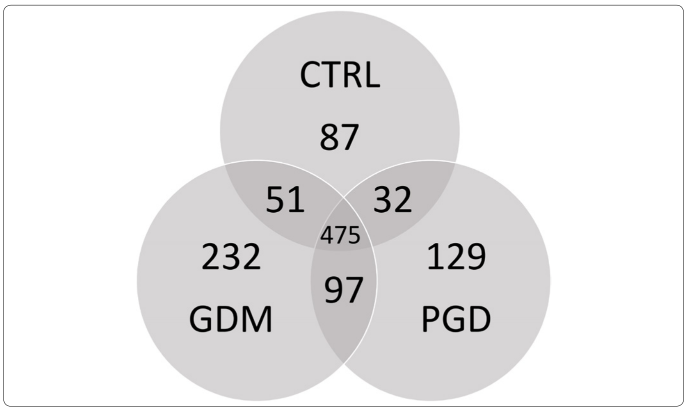

Figure S1: Venn diagram depicting the relationship of 20th week proteins for control (CTRL), gestational diabetic (GDM), and Pregestational diabetic (PGD) proteins. Only proteins present in at least 1 of the 2 replicates of every subject in the group are shown.

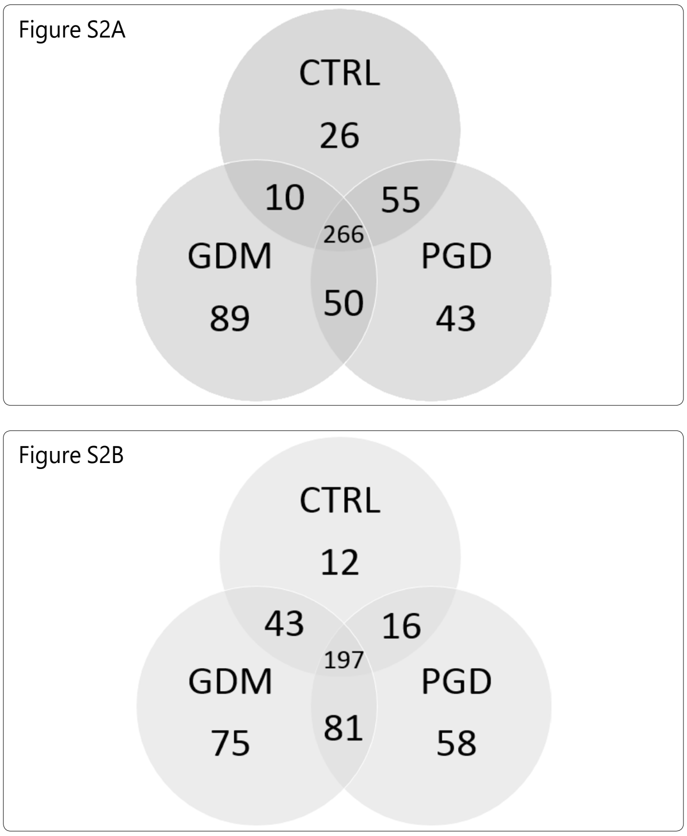

Figure S2: Venn diagram depicting the relationship of 20th week (A) and 36th week (B) proteins for control (CTRL), gestational diabetic (GDM), and Pregestational (PGD) groups.

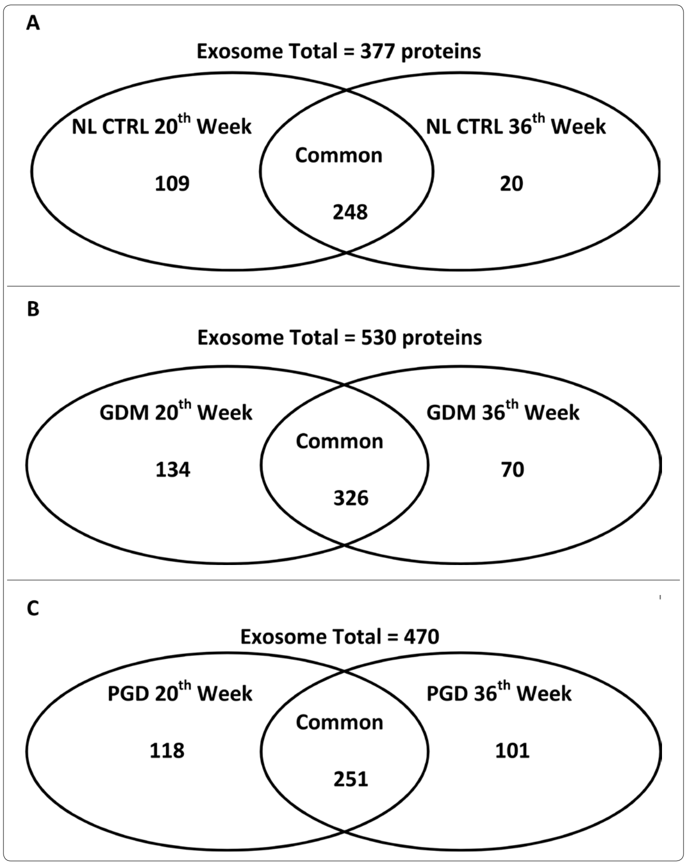

Figure S3: Groupwise 2-way split Venn diagram describing the spread of proteins between 20th and 36th week for CTRL (Panel A), GDM (Panel B) and PGD (Panel C) groups are shown. Whereas more than 5-fold reduction in the number of unique proteins identified between 20th and 36th week is observed in CTRL (109 versus 20); it is approximately half in GDM (134 versus 70), but remains almost unchanged in PGD (118 versus 101).
